# Impact of *Fas/Fasl* Gene Polymorphisms on Susceptibility Risk and Imatinib Mesylate Treatment Response in Chronic Myeloid Leukaemia Patients

**DOI:** 10.31557/APJCP.2021.22.2.565

**Published:** 2021-02

**Authors:** Aziati Azwari Annuar, Ravindran Ankathil, Nazihah Mohd Yunus, Azlan Husin, Nur Shafawati Ab Rajab, Ahmad Aizat Abdul Aziz, Mohd Ismail Ibrahim, Sarina Sulong

**Affiliations:** 1 *Human Genome Centre, School of Medical Sciences, Universiti Sains Malaysia, Kelantan, Malaysia.*; 2 *Department of Medicine, School of Medical Sciences, Universiti Sains Malaysia, Kelantan Malaysia. *; 3 *Department of Community Medicine, School of Medical Sciences, Universiti Sains Malaysia, Kelantan, Malaysia. *

**Keywords:** FAS/FASL, Chronic myeloid leukaemia, promoter polymorphism, imatinib mesylate, resistance

## Abstract

**Background:**

The *FAS* mediated apoptosis pathway involving the *FAS* and FASL genes plays a crucial role in the regulation of apoptotic cell death and imatinib mesylate (IM) mechanism of action. Promoter polymorphisms *FAS-670 A>G *and *FAS-844 T>C* which alter the transcriptional activity of these genes may grant a risk to develop cancer and revamp the drug activities towards the cancer cell. We investigated the association of these two polymorphisms with the susceptibility risk and IM treatment response in Malaysian chronic myeloid leukaemia (CML) patients.

**Methods:**

This is a retrospective study, which included 93 CML patients and 98 controls. The polymerase chain reaction restriction fragment length polymorphism (*PCR-RFLP*) method was used to genotype the *FAS* and* FASL* polymorphisms. Data nanlysis was done using SPSS Version 22. The associations of the genotypes with susceptibility risk and IM response in CML patients were assessed by means of logistic regression analysis and deriving odds ratio with 95% CI.

**Results:**

We observed a significant association between *FASL-844T>C* polymorphism and CML susceptibility risk and IM response. Variant C allele and FASL-844 CC variant genotype carriers had significantly higher risk for CML susceptibility (OR 1.756, CI 1.163-2.652, p=0.007 and OR 2.261, CI 1.013-5.047, p=0.047 respectively). Conversely, the heterozygous genotype FASL-844 TC conferred lower risk for CML susceptibility (OR 0.379, CI 0.176-0.816, p=0.013). The heterozygous and homozygous variant genotypes and variant C alleles were found to confer a lower risk for the development of IM resistance with OR 0.129 (95% CI: 0.034-0.489 p=0.003), OR 0.257 (95% CI: 0.081-0.818, p=0.021), and OR 0.486 (95% CI: 0.262-0.899, p=0.021) respectively. We also found that *FAS-670 A>G* polymorphism was not associated with CML susceptibility risk or IM response.

**Conclusion:**

The genetic polymorphism* FASL-844 T>C* may contribute to the CML susceptibility risk and also IM treatment response in CML patients. Accodringly, it may be useful as a biomarker for predicting CML susceptibility risk and IM resistance.

## Introduction

Chronic myeloid leukemia (CML) is a monoclonal myeloproliferative disorder of hematopoietic stem cells. It strikes about one to two individual per 100, 000 population annually with a slight male preponderance, and accounts for 15% of leukemia in adults of the Western Hemisphere. Before development of the targeted therapy with tyrosine kinase inhibitors (TKIs), the median survival of CML was 5–7 years (Kantarjian et al., 2012).

The Philadelphia (Ph) chromosome, the cytogenetic hallmark of CML, is formed as a result of reciprocal translocation between chromosome 9 and 22 (Kurzrock et al., 1988). The sequel of this translocation eventually causes formation of BCR-ABL fusion oncogene that is implicated in the etiopathogenesis of CML. This BCR-ABL1 oncogene encodes the chimeric BCR-ABL1 protein which harbours constitutive kinase activity. Formation of *BCR-ABL1* fusion protein leads to uncontrolled cell proliferation, making the cells resistance to apoptosis and hence increasing leukaemic stem cell survival. However, evidence shows that Ph chromosome alone is insufficient for the development of CML (Bose et al., 1998). Few earlier studies had demonstrated the presence of very low level of *BCR-ABL* fusion transcripts in the peripheral blood of healthy individuals who never developed CML, implicating that host genetic susceptibility factors are also important in the CML pathogenesis. However, the host genetic susceptibility factors favoring CML development have not been identified yet. Genetic variations such as functional single nucleotide polymorphisms (*SNPs*) in the apoptosis regulatory genes have been shown in the pathogenesis of malignant tumors and autoimmune diseases (Edathara et al., 2016).

Apoptosis occurs through two main mechanisms either a change in the mitochondrial permeability (intrinsic pathway) or binding of the death receptor FAS and its specific ligand FASL to the cell surface (extrinsic pathway) (Ozdemirkiran et al., 2017). FAS gene, (also known as APO-1, apo-1 antigen, CD95, or tumour necrosis factor receptor superfamily number 6/TNFRSF6) is located at long (q) arm of chromosome 10 at position 23.31 based on National Center for Biotechnology Information (NCBI). FAS is a molecule expressed on the cell surface and initiates the death signal after binding to its ligand FASL. The FAS/FASL pathway is a critical system for hematopoietic cell survival and apoptosis(Ozdemirkiran et al., 2017); Fusun et al 2015). 

Binding of FAS receptor to FAS ligand results in the formation of death inducing signaling complex (DISC), which then results in the initiation of apoptosis by cleaving downstream and executioner caspases. Decreased expression in FAS or increased expression of FASL have been detected in many cancers and been associated with aggressive tumor behavior. Reduced expression of FAS may cause less cancer cells to undergo apoptosis and at the same time may protect the cells from elimination by antitumor immune response, while increased FASL expression may increase the ability of tumor cells to counterattack the immune system by killing FAS–sensitive lymphocytes, thereby contributing to cancer development (Gratas et al., 1998). Thus, down regulation of FAS can result in resistance to apoptosis. Genetic variations such as SNPs in FAS may have a contributory sole in altering cancer susceptibility risk, including CML. Even though few studies have shown the association of *FAS/FASL* polymorphisms with susceptibility risk of various cancers, till date there is only one report on its association with CML susceptibility risk worldwide (Edathara et al., 2016). 

Imatinib mesylate (IM) is a selective and specific ABL-tyrosine kinase inhibitor that shows outstanding therapeutic benefits for patients with CML. Even though excellent treatment responses were obtained with IM, a significant proportion of patients develop resistance or show suboptimal response to IM (Au et al., 2014), thereby indicating existence of inter individual variability of response to IM. Clinical responses of IM possibly rely on genetic background of the patient. IM acts by inhibiting the cellular growth and inducing apoptosis (Goldman and Melo, 2003). The apoptotic signaling regulatory genes, FAS/FASL play an important role in mediating apoptosis pathway in this drug’s mechanism of action. IM modulates different anti-apoptotic and proapoptotic proteins belonging to the extrinsic and intrinsic pathways of apoptosis. Since genetic variation in FAS may contribute to down regulation of FAS, inter individual variations, as a result of SNPs, may result in inter patient variability in apoptotic regulation and contribute to drug resistance. Inter individual apoptotic variability as a result of polymorphisms in apoptosis pathway related genes can influence the response to IM in CML patients and can be a possible BCR-ABL independent mechanism mediating resistance. 

It was hypothesized that SNPs of FAS/ASL are associated with susceptibility risk and also IM treatment response in CML patients.

## Materials and Methods

*Study participants*

This retrospective study was approved by Research and Ethics Committee of Universiti Sains Malaysia and Ministry of Health, Malaysia (ethical numbers USMKK/PPP/JEPeM (244.3.(4) and USMKK/PPP/JEPeM (264.3.(8)) and complied with the Declaration of Helsinki. It was undertaken from Oct 2010 until Sept 2013. A total of 93 Ph chromosome-positive BCR–ABL non-mutated CML patients who underwent daily treatment of 400 mg IM for at least 12 months were recruited. They comprised of 47 males and 46 females, predominantly Malay and aged between 11 to 78 years old with the mean age of 41.3. Accordingly, 46 were IM good responders and 47 were IM resistant Ph positive CML patients. All 93 CML patients were included in FAS study, while only 90 patients (45 good IM responders and 45 resistant IM treatments) were included in FASL study. 

These CML patients were selected from two university hospitals in Malaysia, namely Hospital Universiti Sains Malaysia (Hospital USM) and Universiti Kebangsaan Malaysia Medical Centre (PPUKM), and also from few hospitals under the Ministry of Health Malaysia such as Hospital Raja Perempuan Zainab II (HRPZII) Kota Bharu, Kelantan, Hospital Pulau Pinang, Penang and Hosp Sultanah Aminah, Johor Bahru. Control group consisted of 98 healthy individuals from community and Universiti Sains Malaysia healthy staff. They were free from well known diseases and cancer. Two groups were matched in terms of age and gender.Written informed consent was taken from study participants prior to their inclusion in the study. 

*Selection of patients based on IM response*

The haematological, cytogenetics, and molecular responses of all CML patients were assessed by physicians and medical oncologist in each hospital in order to justify the clinical response to IM based on European Leukemia Net (Baccarani et al., 2015) and National Comprehensive Cancer Network (NCCN) (2018). Molecular response was categorized into major molecular response (MMR) and complete molecular response (CMR) based on BCR/ABL fusion and control gene transcript ratios. Patients were categorized under MMR when they showed BCR–ABL transcript level < 0.1% on the international scale. The CMR was characterized by nondetectable BCR–ABL using reverse transcriptase quantitative polymerase chain reaction (RT- Q-PCR) and also transcript level of 0.01%, 0.0032%, or 0.001% depending on the sensitivity of the assay. Cytogenetic response was assessed based on GTG-banded analysis of a minimum of 20 bone marrow metaphases. Cytogenetic response was classified as complete (0% Ph+ metaphases), partial (>0 to 35% PH+ metaphases), minor (>35 to 65% Ph+ metaphases), minimal (>65 to 95% Ph+ metaphases), and no cytogenetic response (>95 to 100% Ph+ metaphases). For haematological response, CML patients were classified as having complete hematological response (CHR) when their platelet count was <450x109/L, WBC <10x109/Land, and basophils count <5%. Patients with a partial cytogenetic response in three months, a complete cytogenetic response in six months, and an MMR in 12 months from the initiation of treatment were considered as IM good responders. Patients who did not achieve the above response criteria within the specified time frame were categorized under IM resistant groups. 

*Genotyping*

The extracted DNA from peripheral blood was used for the amplification of the target sequences of FAS−670 A>G and FASL−844 T>CSNPs by sequence-specific primers. The primer sequences and conditions are mentioned in Table 1. The PCR reaction was carried separately for FAS and FASL. PCR amplification was carried out in a total of 25µl reaction mix. The PCR products were subjected to PCR-restriction fragment length polymorphism (*RFLP*) analysis using respective restriction enzymes and followed by genotyping on 2% agarose gel electrophoresis. Few of the randomly selected representative samples were sent for sequencing to First Base Labs Kuala Lumpur. 

*Statistical analysis*

The genotypes were categorized into homozygous wild type, heterozygous, and homozygous variant. The difference in the genotype frequencies between the two groups was determined using chi square tests. The association of the *FAS* and *FASL* genotypes with CML susceptibility risk and IM treatment response was assessed by means of logistic regression analysis and deriving odds ratio (ORs) with 95% CI. An OR ratio value more than 1 was considered as having higher risk. Homozygous wild genotype, which by definition has an OR=1, was used as reference. A p value less than 0.05 was considered significant for all statistical analyses. Data were analyzed using SPSS Version 22.

## Results

*Genotype and allele frequencies of FAS-670 A>G and FASL-844 T>C polymorphisms in CML patients and controls*

The genotype and allele frequencies of FAS -670 A>G and FASL -844 T>C in CML patients and controls are shown in Table 2. Majority (58.1%) of CML patients showed heterozygous genotype AG. Among the 90 normal controls, 47.8% had heterozygous AG genotype, 33.3% had variant genotype GG, and 18.9% had homozygous wild type AA genotype. Variant allele G was observed in 57.0% and 57.2% of CML patients and controls, respectively. However, there was no significant difference between CML patients and controls regarding the genotype and allele frequencies of *FAS-670 A>G* polymorphism . 

In the case of *FASL-844 T>C *polymorphism studied in 90 CML patients, 50% were detected to have variant genotype CC, 25.6% with heterozygous genotype TC, and 24.4% with homozygous wild type TT. The heterozygous (TC) genotype was significantly higher in controls; whereas, homozygous variant genotype (CC) was significantly higher in CML patients. The variant allele C also was significantly higher in CML patients compared to controls.

*Association of FAS -670 A>G and FASL -844 T>C with CML susceptibility risk*

For *FAS-670 A>G* polymorphism, both heterozygous genotype AG and variant genotype GG showed higher risk values, but causing no statistically significant difference (Table 2). A significant association between *FASL−844T>C* polymorphism and CML susceptibility risk was observed. Both variant C allele and variant genotype CC carried significantly higher risk for CML development; whereas, the heterozygous genotype TC conferred significantly lower risk for CML susceptibility (Table 2).

*Genotype and allele frequencies of FAS-670 A>G and FASL-844 T>C polymorphism in IM resistant and responsive CML patients*

Genotype frequencies of the studied FAS and FASL polymorphisms in IM resistant and response group of CML patients are shown in Table 3. Compared to IM resistant group of CML patients, the heterozygous genotype AG was significantly higher in IM response group. However, there were no significant differences in the frequencies of other genotypes of *FAS-670 A>G *polymorphism between the IM resistant and response group of CML patients.

For *FASL -844 T>C* polymorphisms, the homozygous wild type genotype TT was significantly higher in IM resistant group; whereas, the heterozygous TC genotype was significantly higher in the IM good response group. The allele C was significantly higher in IM good response group.

*Association of FAS-670 and FASL -844 polymorphisms with IM treatment response*

The association of the genotypes and alleles with response to IM treatment in CML patients was determined and the results are shown in Table 3.

With regard to FAS, the variant allele G of *FAS-670 A>G* polymorphism showed a slightly higher risk but statistically insignificant for the development of resistance to IM. The heterozygous genotype AG showed lower risk, but it was not statistically significant. The variant genotype GG also showed no significant difference in risk level compared to the reference homozygous wild type AA.

For FASL-844 T>C, both heterozygous TC and variant CC genotypes showed a significantly lower risk for IM. The variant allele C also was associated with a significantly reduced risk for the development of resistance.

**Table 1:** PCR Conditions Used for *FAS* and *FASL* RFLP Analysis

SNP Polymorphism	Primer sequences	Annealing temperature	Restriction Enzyme used	Product size
*FAS*-670 A>G (rs1800682) (10q23.31)	F 5’-CTACCTAAGAGCTATCTACCGTTC-3’ R 5’-GGCTGTCCATGTTGTGGCTGC-3’	61˚C for 30s	BstN1	A allele 231 and 101bp G allele 184,101 and 47bp
*FASL*-844 T>C (rs763110) (1q24.3)	F 5’-GAAGCTAGGAGTTTGAGACAAG-3’ R 5’-ACACAACTACCATTTACCCTGA-3’	64.4˚C for 30s	BSrD1	T allele 274 and 132bp C allele 406bp

**Table 2:** Genetic Association of *FAS*-670 A>G and *FASL* -844 T>C Polymorphisms with CML Susceptibility Risk

*FAS* -670 A>G	CML patients (n=93)	Controls (n=90)	P value	Adjusted OR (95% CI)	P value
Genotype					
AA	13 (13.9%)	17 (18.9%)	0.37	1 (Reference)	
AG	54 (58.1%)	43 (47.8%)	0.163	1.642 (0.719-3.750)	0.239
GG	26 (27.9%)	30 (33.3%)	0.43	1.133 (0.464-2.768)	0.784
Allele					
A	80 (43.0%)	77 (42.8%)		1 (Reference)	
G	106 (57.0%)	103 (57.2%)	1	0.991 (0.655-1.499)	1
*FASL* -844 T>C	CML patients (n=90)	Controls (n=98)	P value	Adjusted OR (95% CI)	P value
Genotype					
TT	22 (24.4%)	21 (21.4%)	0.624	1 (Reference)	
TC	23 (25.6%)	58 (59.2%)	<0.001*	0.379 (0.516-0.816)	0.013*
CC	45 (50%)	19 (19.4%)	<0.001*	2.261 (1.013-5.047)	0.047*
Allele					
T	67	100		1 (Reference)	
C	113	96	0.007*	1.756 (1.163-2.652)	0.007*

*p<0.05 statistically significant; OR, odd ratio; CI, confidence interval

**Table 3:** Genetic Association of *FAS*-670 A>G and *FASL* -844 T>C Polymorphisms with IM Response in CML Patient

*FAS* -670 A>G	Treatment status (n=93)				
	Resistance (n=46)	Response (n=47)	P value	Adjusted OR (95% CI)	P value
Genotype					
AA	8 (17.4%)	5 (10.6%)	0.348	1 (Reference)	
AG	22 (47.8%)	32 (68.1%)	0.048*	0.429 (0.124-1.488)	0.43
GG	16 (34.8%)	10 (21.3%)	0.147	1.000 (0.255-3.929)	1
Allele					
A	38 (41.3%)	42 (44.7%)		1 (Reference)	
G	54 (58.7%)	52 (55.3%)	0.639	1.148 (0.642-2.052)	0.639
*FASL*-844 T>C	Treatment status (n=90)				
	Resistance (n=45)	Response (n=45)	P value	Adjusted OR (95% CI)	P value
Genotype					
TT	17 (37.8%)	5 (11.1%)	0.003*	1 (Reference)	-
TC	7 (15.5%)	16 (35.6%)	0.029*	0.129 (0.034-0.489)	0.003*
CC	21 (46.7%)	24 (53.3%)	0.527	0.257 (0.081-0.818)	0.021*
Allele					
T	41 (45.6%)	26 (28.9%)		1 (Reference)	
C	49 (54.4%)	64 (71.1%)	0.002*	0.486 (0.262-0.899)	0.021*

*p<0.05 statistically significant; OR, odd ratio; CI, confidence interval

## Discussion

An important anti-carcinogenic pathway is apoptosis in all cell types, the evasion of which is one of the hallmark of human cancers (Hanahan and Weinberg, 2011). Apoptosis is most commonly initiated through the death receptor pathway. The most well characterized death receptor is the Fas receptor, which is encoded by FAS. Promoter polymorphisms in the *FAS* influence its transcriptional regulation (Shimonishi et al., 2000; Tan and Ankathil, 2015). Down regulation of FAS can result in resistance to apoptosis and also to drugs. Although numerous studies have investigated the association of *FAS* polymorphisms with different types of cancer, limited data are available on their possible involvement in susceptibility risk and IM treatment response in CML patients. 

In general, many previous studies demonstrated that increased expression of FASL and reduced expression of FAS were usually signs of malignant transformation and early features of development of human cancers (Gratas et al., 1998).Variety of tissues express FAS receptor; whereas, FASL receptors are only found in certain cells, mainly the cells involved with the immune system such as activated T cells and natural killer cells (Zhang et al., 2006). The tumour cells generally overexpress Fas ligand. This is considered as a tumour counter attack mechanism which eventually will induce the apoptosis of infiltrating lymphocytes, allowing the tumour to escape from the effects of immune response (O’Connell et al., 1999). 

Fas ligand (FasL, Apo-1,CD 178) is a key apoptosis inducing ligand of the TNF family death factors (Yonehara et al., 1989). Once FASL is bound to its receptor FAS, (Apo-1, CD95) it will induce apoptosis via a death receptor signaling pathway. This trimerization later triggers caspase 8 and 10. The caspases 8 and 10 are known as initiator caspases. Subsequently, these initiator caspases stimulate apoptosis by activating executive caspase 3 directly, or by activating BID protein and releasing cytochrome C from mitochondria (Wang et al., 2010).

Wu et al., (2003) reported that the *FASL-844 T>C *polymorphism was located at the promoter region in a binding site for CAAT enhancer protein. They also reported that C allele of this polymorphism had a threefold increased binding capacity to the CAAT enhancer protein and hence increased the *FASL* expression. Since expression of FASL was restricted mainly to the activated T cell and natural killer cells, it is reasonable to suggest that high level of FASL may create an immune privileged site by killing cytotoxic immune cells and therefore escaping host immune mechanism of protection, thus influencing cancer risk. These results were found to be in agreement with a previous study (Edathara et al., 2016) which reported that FASL-844 CC and FASL-844 TC genotypes conferred significant risk for developing CML in Indian population. Very few reports are available on this *FASL *polymorphism and its association with CML susceptibility risk. However, there are few other studies which reported that *FASL-844T>C* polymorphism significantly increased the risk of lung cancer (Zhang et al., 2005), oesophageal cancer, (Sun et al., 2004a), ovarian cancer (Li et al., 2013), and Systemic Lupus Erythematous (Wu et al, 3003). On the contrary, no significant association betwwen *FASL-844* polymorphisms and development of leukaemia (Chen et al., 2015), breast cancer (Sun et al., 2004a), and chronic myeloproliferative disorder (Ozdemirkiran et al., 2017) have been reported. 

For *FAS-670 A>G* polymorphism, both heterozygous genotype AG and variant genotype GG also showed higher risk values, but causing no statistically significant difference. Lack of statistical significance could be attributed to the small sample size in the present study. A previous study by Edhatara et al., (2016) on 772 patients from India also found that variant genotype GG of FAS-670 was associated with increased risk of CML development. These findings were also in concordance with earlier studies carried out on lung cancer (Zhang et al., 2005; Wang et al., 2003), esophageal squamous cell carcinoma (Sun et al., 2004b), chronic myeloproliferative disorder (Ozdemirkiran et al., 2017), and breast cancer (Hashemi et al., 2013). It has been suggested that the transition of FAS-670 from A to G in the promoter region of signal transducer and activator of transcription (STAT) 1 protein binding element eventually diminishes the promoter activity and decreases FAS expression (Huang et al., 1997), which in turn reduces the apoptosis of abnormal cells and increases the carcinogenesis risk. Tumorigenesis is closely related to resistance to apoptosis and development of CML as it allows malignant cells to grow even in a stressful environment. However, (Park et al., (2006) showed no significant association between *FAS-670 A>G* polymorphism and lung cancer in Korean population. 

Regarding the genotype of the control population, our study demonstrated that most of the control population showed FAS-670 AG heterozygous genotype. This was in agreement with a study by Edathara et al., (2016) and other studies done on other types of leukaemia in patients with Caucasian, Asian, and Latinas ethnicity (Chen et al, 2015). With respect to FASL-844, we found that majority of the control population showed FASL-844 TC heterozygous genotype with predominant T allele. This finding was in line with that reported byTong et al., (2012) which was done in China on acute lymphoblastic leukaemia.

The FAS/FASL mediated apoptosis pathway is important in IM mechanism. Once IM binds to the BCR-ABL oncoprotein, the kinase activity is inhibited and the subsequent signal transduction pathway that results in apoptosis is inactivated. At the same time, IM was found to have the ability to restore the proapoptotic proteins, mainly BIM, that were inhibited by the BCR-ABL fusion protein. In an earlier study, it was established that the induction of BIM was a key step in the induction of apoptosis in the hematopoietic cells (Kuroda et al., 2006). Cells resistant to apoptosis are also predicted to be resistant to cancer therapy (de Bruin and Medema, 2008). However, our findings showed that patients with the FASL-844 TC and FASL-844CC genotypes had significantly reduced risk of resistance towards IM therapy. We also found that the variant C allele significantly had a lower risk against development of resistance to IM therapy. Our finding is consistent with a previous study by Zheng et al., (2016) who found that carriers of FASL-844 CC genotype had higher major molecular response compared to those carrying TT/TC genotypes. 

The FASL-844 T>C SNP is located within a putative binding motif in the CAAT/enhancer-binding protein β transcription factor. Higher expression of FASL is associated with the C allele compared to the T allele (Wu et al, 2003). Thus, the polymorphism may affect FASL expression followed by subsequent *FAS/FASL* signaling, leading to apoptosis of cancer cells (Mahfoudh et al., 2012). Edathara et al., (2016); however, revealed no significant association between *FASL* polymorphism and IM therapy. Another study on Chron’s disease found that FASL TT genotype was associated with reduced response to infliximab therapy for Chron’s disease (Hlavaty et al., 2005).

In this study, we found no significant correlation between *FAS-670 A>G* polymorphism and IM treatment response. This could be attributed to the small sample size in our study. The G allele might result in lower FAS expression and lower apoptosis of leukemic cells, thus contributing towards disease progression and development of resistance to IM. The A to G transition of FAS-670 in the enhancer region has been reported to modify the signal transducer and activator of transcription, reduce the activity of promoter region, and finally down regulate FAS expression. Edathara et al., (2016) reported that none of the polymorphisms of *FAS*, either* FAS-670* or *FAS-3770*, were associated with haematological and cytogenetic response in 386 CML patients in India. In contrast, Zheng et al., (2016) noted a significant association between FAS-670 GG genotype and complete molecular response in 187 Korean CML patients. The inconsistency of the present result with those of other results could be attributed to the difference in genetic background of the study subjects, difference in phases of CML either chronic, accelerated, or blast phase, and difference in sample sizes.

One of the limitation of the present study was that we examined the association between only two SNPs FAS/FASL and the susceptibility risk and IM response in CML patients. Other SNPs or genes that are involved in the cell death pathway which might be relevant to CML susceptibility risk and IM response were not covered in this study and need to be investigated in further studies. Such studies may help improve our understanding regarding the role of *FAS/FASL* polymorphism in CML susceptibility risk and development of resistance to IM. This in turn may help in better prognosis and management of CML patients.

Our study demonstrated an association between genetic polymorphisms in apoptotic pathway gene *FASL-844 T>C* but not *FAS-670 A>G* and the susceptibility risk as well as treatment response towards IM in Malaysian CML patients. Although a prospective study with a larger patient population is necessary to validate these findings, detection of *FASL-844 T>C* may predict a higher risk of CML development and may also help in predicting the response to IM in CML patients.
